# An easy method for processing and identification of natural and synthetic microfibers and microplastics in indoor and outdoor air

**DOI:** 10.1016/j.mex.2019.11.032

**Published:** 2019-12-04

**Authors:** Joana C. Prata, Joana L. Castro, João P. da Costa, Armando C. Duarte, Mário Cerqueira, Teresa Rocha-Santos

**Affiliations:** a Centre for Environmental and Marine Studies (CESAM) & Department of Chemistry, University of Aveiro, 3810-193 Aveiro, Portugal; b Centre for Environmental and Marine Studies (CESAM) & Department of Environment and Planning, University of Aveiro, 3810-193 Aveiro, Portugal

**Keywords:** Method for sampling microfibers and microplastics in air, Atmospheric contamination, Particulate matter, Synthetic textile fibers, Natural textile fibers

## Abstract

Microplastics and microfibers can contaminate every matrix, including in the atmosphere, thus leading to incidental inhalation. However, concentrations of airborne synthetic particle in indoor and outdoor environments are not well understood due to the complexities of sampling, sample processing and identification. This work aims at producing a simple protocol to determine the concentrations of airborne microplastics and fibers. This is accomplished by removing organic matter using hydrogen peroxide (H_2_O_2_), followed by removal of mineral matter by density separation with sodium iodide (NaI). Finally, identification of fibers into synthetic or natural under the stereomicroscope can be achieved following a diagram produced by systematically observing the most common textile fibers. This method produces a recovery rate of 94.4 % for spiked samples and has been proven suitable for environmental samples.

• *Fibers and microplastics in air are easier to identify after carbonaceous matter removal;*
• *No loss of microfiber is expected from the solutions used;*
• *Recovery rates of spiked samples is 9*4.4 %.

## Graphical abstract

**Figure:**
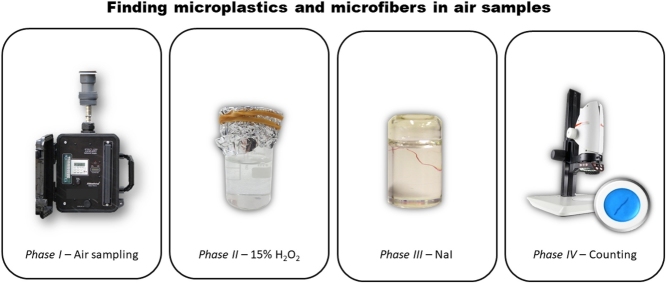

**Specification Table**

**Table:** 

Subject Area:	Environmental Science
More specific subject area:	Air pollution
Method name:	Method for sampling microfibers and microplastics in air
Name and reference of original method:	Abbasi S., Keshavarzi B., Moore F., Turner A., Kelly F.J., Dominguez A.O., Jaafarzadeh N. (2019) Distribution and potential health impacts of microplastics and microrubbers in air and street dusts from Asaluyeh County, Iran. Environmental Pollution 244, 153-164. https://doi.org/10.1016/j.envpol.2018.10.039
Resource availability:	• Metal tweezers; • Glass beakers; • Glass Petri dish; • Glass pipette; • Glass filtration system; • Filtration pump; • Aluminum foil; • Quartz fiber filters (Whatman QMA); • Glass fiber filters (Whatman GF/C^TM^); • Sterile gloves (Naturflex® Powder-free Nitrile, Bimedica). Solutions: • Ultrapure water (Elix®); • 30 % hydrogen peroxide (H_2_O_2_) (Labkem); • 1.6 g cm^−3^ sodium iodide (NaI) (Sigma-Aldrich). Equipment • Portable active air samplers (AirMetrics model MinVol^TM^ TAS); • Laminar flow hood; • Vortex; • Stereomicroscope (Leica DMS300). Software • ImageJ.

## Method details

Microplastics and microfibers, deriving from modern products and activities, are released to the atmospheric compartment [1]. Currently, only 4 works have been published sampling airborne microplastics and microfibers by active sampling [[1], [2], [3], [4]]. These works rely on visual identification and chemical characterization by micro-Fourier transform infrared spectroscopy (micro-FTIR). However, chemical identification by spectroscopic methods, such as micro-Raman spectroscopy or micro-FTIR, is time consuming and not always available [5]. Visual characterization of microfibers as synthetic or natural is difficult, especially when lacking concrete parameters. Although identification of microplastics can be aided using staining dyes, namely Nile Red, and the use of automated software, such as MP-VAT, individualized fibers do not present fluorescence following current staining protocols [6]. Identification is even more complex in the presence of organic and mineral contaminants.

Sampling of passive deposition of atmospheric particles and collection of street dust rich in organic matter led to the development of a method including organic matter removal and density separation [7]. However, the original protocol was complex as it required several passages and drying steps that consumed a lot of time and increased the possibility of contamination. Instead, this protocol was simplified and the number of steps reduced. Shortly, air sampling is conducted over a relevant period of time, in this case 48 h (*phase 1*), followed by sample transfer by washing of the quartz fiber filters to glass beakers where H_2_O_2_ is added to achieve a concentration of 15 % and left to react for 8 days to allow removal of natural organic matter (*phase 2*). This solution is then filtered and the sample transferred again to allow density separation through the use of 1.6 g cm^−3^ NaI, removing higher density particles such as inorganic matter (*phase 3*), finally followed by filtration, drying and manual counting under a stereomicroscope following a comprehensive diagram that aids the visual classification of fibers into natural or synthetic (*phase 4*). The suitability of this protocol was then tested using spiked samples, with known numbers, and with indoor and outdoor particulate matter samples. The objective of this protocol was the removal of organic matter and dark particles coating the filter, likely comprised of carbonaceous mater, that hindered quantification and characterization of microfibers and suspected microplastics.

## Prevention of contamination

Prevention of contamination and blanks are especially important when handling air samples since it will mostly be comprised of settling fibers. The first step in preventing contamination is using exclusively glass and metal materials, refusing the use of plastics, and previously washing all materials with HNO_3_ and distilled water, and covering them with aluminum foil. Right before use, materials were passed again in HNO_3_ and distilled water, and covered with paper towels. The filtration system was thoroughly washed between samples with distilled water. All solutions were filtered beforehand and kept in glass bottles sealed with aluminum foil caps. Only Elix® ultrapure water was used. All filters, of analytical grade, were fired at 475 °C for 3 h in the muffle furnace to remove fibers and contaminants. Work was conducted under a laminar flow hood, previously cleaned with a hand duster followed by cleaning with 70 % ethanol in a paper towel. Counting of samples was also conducted inside the laminar flow hood using a previously cleaned digital stereomicroscope. A cotton lab coat was worn at all the times. Whenever possible, sleeves were pulled back to avoid the release of fibers (even of cotton ones) inside the laminar flow hood and the arms and hands washed following surgical scrub procedures. When handling H_2_O_2_ and NaI solutions, sleeves were pulled down and sterile gloves, straight from the packaging, worn. Petri dishes, in which samples were kept, were washed with HNO_3_, distilled water, dried and then the remaining fibers cleaned using an air jet. Petri dishes were kept closed as much as possible to avoid contamination. For weighting, filters were loaded into pre-weighted Petri dishes in the laminar flow hood, which were then weighted in the analytical scale. Solutions in beakers were also covered with aluminum foil and only opened when strictly necessary. Two procedural blanks were subjected to the same procedures as samples, and an open Petri dish containing a clean filter allowed to evaluate potential contamination inside the laminar flow hood.

### Phase 1 – indoor and outdoor air sampling

Airborne particles were concentrated on quartz fiber filters using portable active samplers (AirMetrics model MinVol™ TAS) equipped PM_10_ size selective inlets. Sampling was performed at a flow rate of 5 L min^−1^ over a 48 -h period. Quartz fiber filters were then removed with metal tweezers, stored in sealed clean glass Petri dishes. This protocol was used for indoor and outdoor sampling.

### Phase 2 – removing organic matter with 15 % H_2_O_2_

**Fig. 1:**
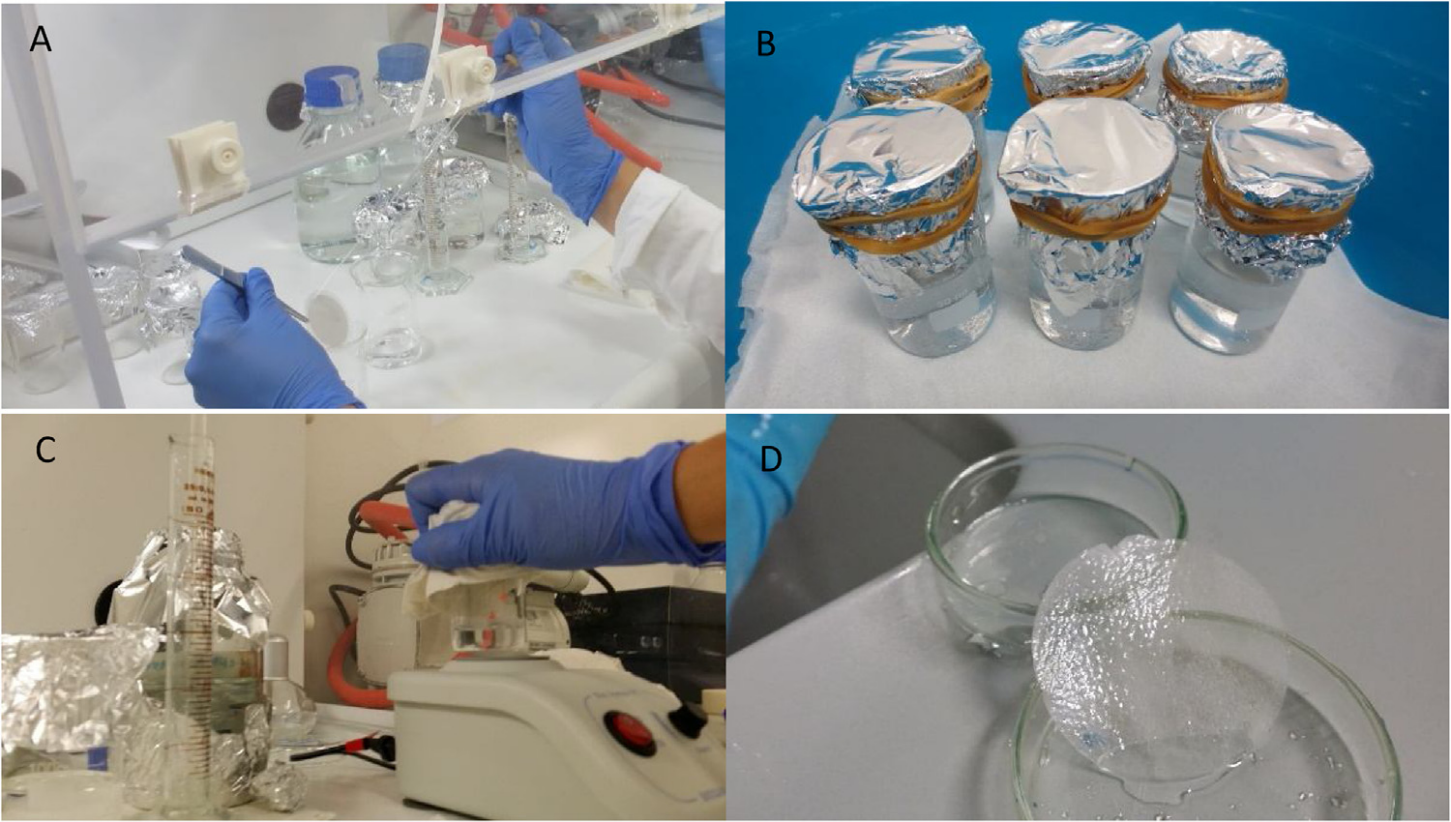
Images of procedures being conducted in the laminar flow hood: (A) transferring samples from the original quartz fiber filters with distilled water, after which H_2_O_2_ is added to reach a concentration of 15 %; (B) air samples in 15 % H_2_O_2_ are left to react over the counter for a week, properly sealed with aluminum foil; (C) after a second transfer to a saturated solution of NaI, samples are shaken in the vortex for 1 min and left to settle for 90 min before being filtered; (D) glass fiber filter after being washed with NaI loses its integrity.

After sampling, particulate matter was transferred from quartz fiber filters to an aqueous solution. In a laminar flow hood, filters held by metal tweezers over a glass beaker were washed with 15 mL of ultrapure water using a glass pipette. The Petri dish was also washed with these 15 mL in order to collected loose fibers that might have fallen from the filter during transportation (Fig. 1). To these 15 mL of water containing the sample, 15 mL of previously filtered 30 % H_2_O_2_ was added in order to reach a final concentration of 15 % H_2_O_2_. Glass beakers were covered with aluminum foil and left to react for 8 days at room temperature (20 °C).

### Phase 3 –density separation with NaI

After 8 days of organic matter removal, the solution was filtered in a glass fiber filter, the beaker washed with abundant ultrapure water to recover the remaining sample, and the filtration system cup thoroughly washed with ultrapure water to recover samples possibly adhering to its glass walls. Samples in filters were then transferred to a NaI solution for density separation as previously described, by holding filters by metal tweezers over glass beakers and washing them with 10 mL of NaI. Glass beakers covered with aluminum foil caps were shaken in the vortex for one minute, to separate fibers and plastics from contaminants, and left for 90 min to settle (Fig. 1). The remaining solution was carefully decanted into a new glass fiber filter in the filtration system, without resuspending sedimented samples, followed by filtration of ultrapure water to remove the remaining NaI from the filter.

### Phase 4 – quantification and classification of microfibers

**Fig. 2:**
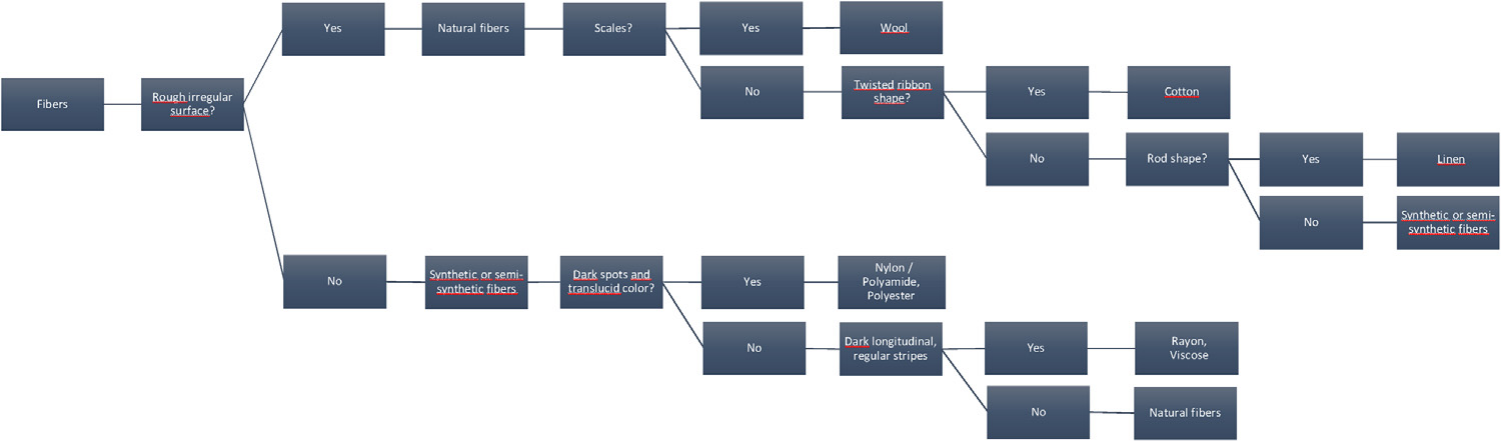
Diagram of the major longitudinal characteristics of different types of textile fibers.

**Fig. 3:**
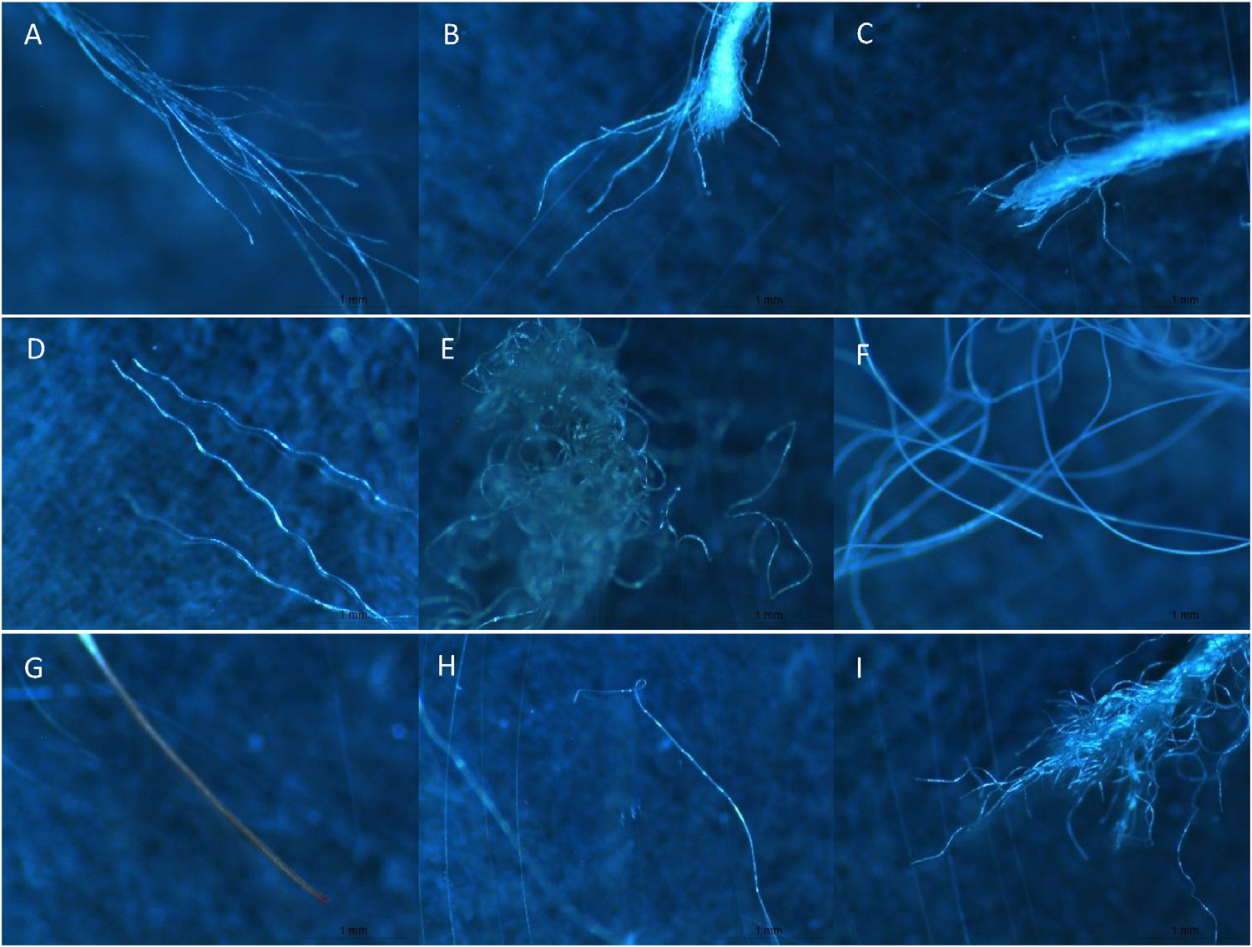
Digital spectroscopy photography of the morphological characteristics of common textile fibers: (A) cotton; (B) linen; (C) cotton polyester blend; (D) polyester; (E) nylon; (F) polyamide; (E) wool; (G) rayon; (H) viscose.

Finally, glass fiber filters in glass Petri dishes were dried over the counter and counted under the stereomicroscope. Samples were opened strictly when necessary, to avoid contamination. Photographs were taken using a digital stereomicroscope and used for measuring fiber length using ImageJ free software and classification regarding color (light, dark). Fibers and microplastics were counted manually. Classification of fibers into natural or synthetic was done manually following the classification diagram presented in Fig. 2, created after visualization and classification of fibers from the most common textile types (Fig. 3). Generally, rough irregular fibers are of natural origin, while synthetic or semi-synthetic present regular surfaces. This classification is also supported by forensic sciences, where natural fibers have generally less regular appearance, with vegetable fibers being flat and twisted and animal fibers presenting surface scales, while synthetic fibers present smooth or regular striated surfaces [8]. In addition to the visual identification conducted, this protocol could be coupled with the use of Nile Red (for microplastics) or micro-Raman spectroscopy.

## Assessing suitability of the protocol

To assess the efficiency of the protocol, three spikes were prepared, each containing 3 polystyrene fibers (Polystyrene Standard 2,000,000, Fluka, Chemica) and 3 polyethylene fragments (grounded Polyethylene, Sigma-Aldrich), which were previously kept in 1 % sodium dodecyl sulfate (Sigma-Aldrich) to improve surface tension. These synthetic polymers were preferred to textile fibers, which were difficult to individualize. Spiked samples were then processed similarly to air samples. All particles were recovered except for one polyethylene fragment, translated in an average recovery rate of 94.4 % (Fig. S-1). This missing particle was later found in the inside of the respective NaI beaker, probably by adhering to the glass wall during decantation.

**Fig. 4:**
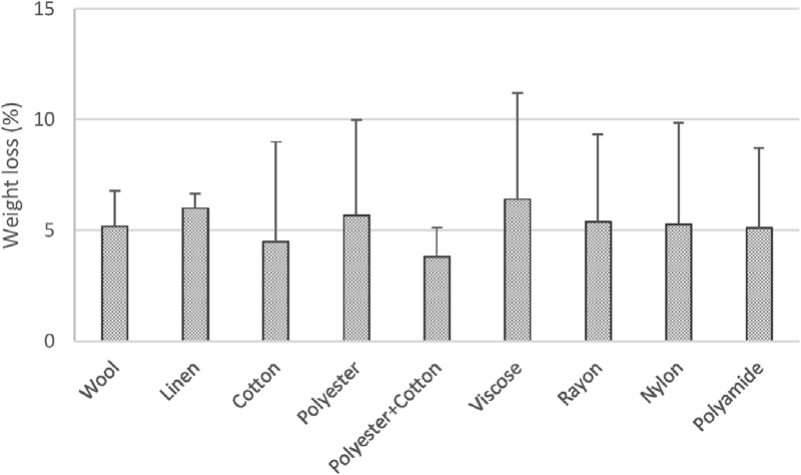
Weight loss of common textile fibers exposed to 15 % H_2_O_2_ for 8 days at room temperature (20 °C).

To assess the suitability of the organic matter removal method, wool, linen, cotton, polyester, polyester with cotton, viscose, rayon, nylon, polyamide, obtained from a market and properly characterized by microscopy and FTIR, were cut into pieces and exposed in three replicates to 15 % H_2_O_2_ for 8 days at room temperature. These common textile fibers lost less than 10 % of their weight (Fig. 4), a variation likely caused by their low initial weight, revealing that this procedure is unlikely to cause fiber loss, even for natural fibers (*e.g.* cotton), during *Phase 2*.

**Fig. 5:**
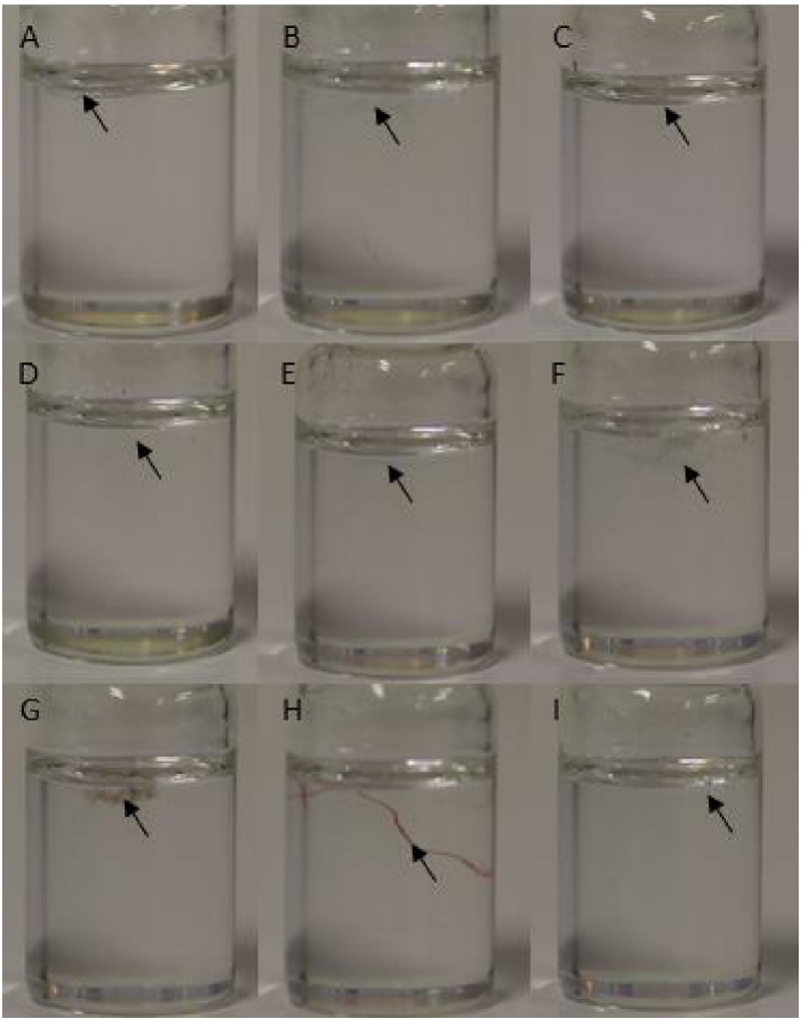
Fibers of cotton (A), polyester (B), cotton polyester blend (C), linen (D), viscose (E), polyamide (F), nylon (G), wool (H) and rayon (I) in a saturated solution of NaI (1.6 g cm^−3^).

Density separation with NaI (1.6 g cm^−3^) could also induce some loss of fibers and plastics. However, plastics density ranges from 0.8 to 1.6 g cm^−3^ [9] and textile fibers from 1.1 to 1.6 g cm^−3^ (Table 1). To confirm this data, textile fibers were placed in a flask containing a NaI saturated solution, covered and vigorously shaken, and then photographed over the counter. Indeed, common textile fibers float in the presence of NaI (Fig. 5). Moreover, density separation using NaI has been shown efficient in the separation of high-density plastics from higher density particles, such as mineral matter [10]. Thus, no loss is expected during the density separation process in *Phase 3*.

**Table 1:** Density of synthetic, semi-synthetic and natural textile fibers, adapted from Morton and Hearle [11].

Fiber	Density (g cm^−3^)
Polyamide / Nylon	1.14
Acrylic	1.19
Silk	1.34
Wool	1.30
Polyester / Polyethylene terephthalate	1.39
Viscose / Rayon	1.52
Cotton / Linen	1.55

## Using the protocol for real indoor and outdoor air samples

**Fig. 6:**
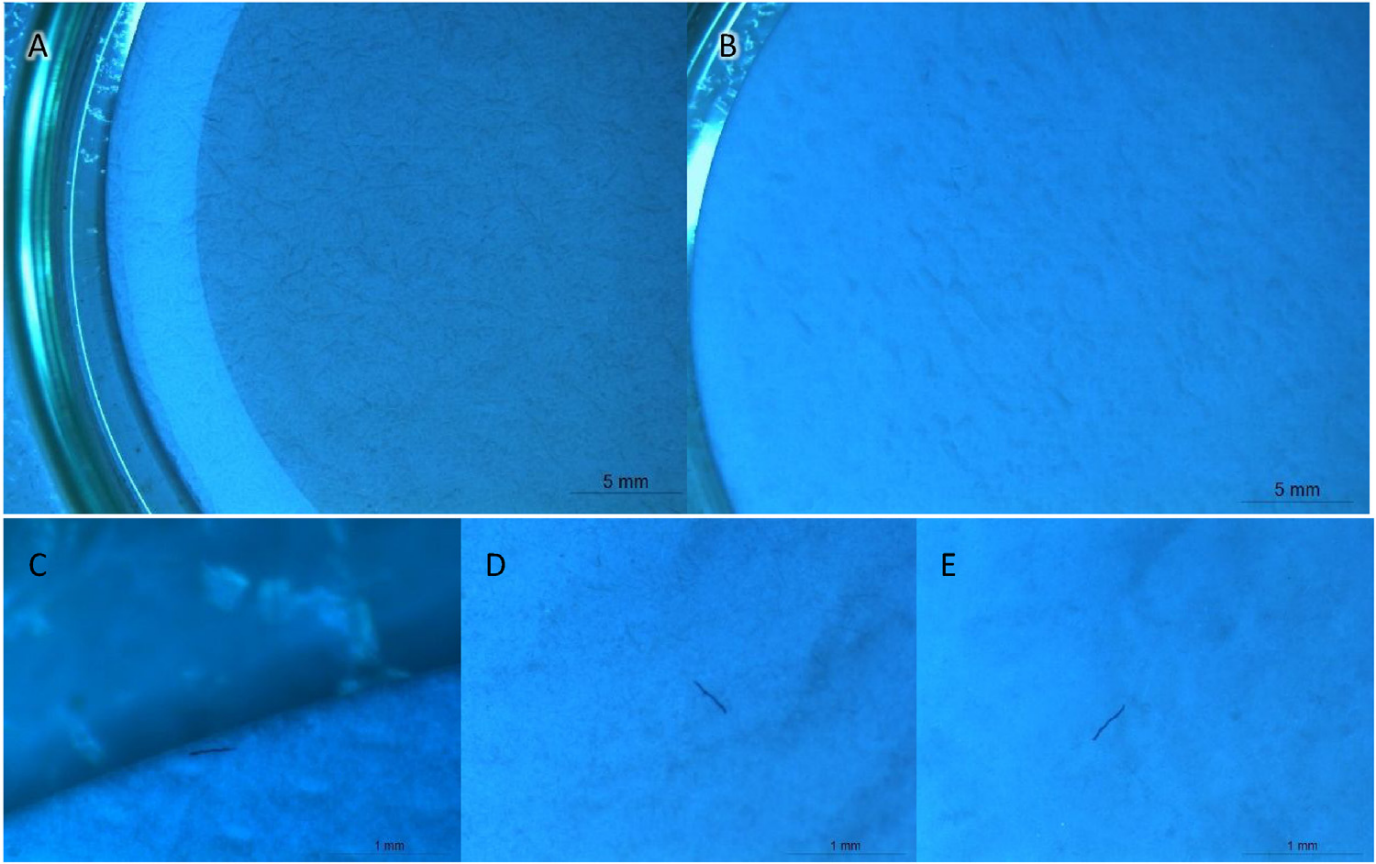
Original quartz fiber filter (A) and after organic matter removal and density separation, at the end of the process (B). Dark fiber in the original quartz fiber filter (C), after 15 % H_2_O_2_ organic matter removal (D), and after NaI density separation (E).

Use of the protocol in environmental samples produced good results, with the removal of the contaminant organic and mineral matter that aided the identification of fibers and microplastics (Fig. 6). Both transfers of sample to H_2_O_2_ and NaI took an average of 20 min per sample. In the stereomicroscope, under 6x, observation and photography of fibers in a full 47 mm filters without landmarks was difficult, taking 30 min per sample. Afterwards, fibers in photographs were characterized in ImageJ regarding size, color (light, dark) and origin (natural, synthetic, unknown), taking at least 1 h per sample. The abundance of fibers and particles resulted from the long sampling time of 48 h in *Phase 1*, which could be shortened into relevant periods of time to reduce the amount of sample. This protocol can be successfully reproduced under 11 days, considering two days for sampling, 8 days for H_2_O_2_ and a final day for density separation, drying, and counting filters under the stereomicroscope.

Fibers were observed more easily after the protocol, as most of the carbonaceous matter covering their surface was removed, revealing a higher number of light-colored fibers than expected from observation of the filters before any treatment (37 % before *vs.* 72 % after). Fiber sizes presented an average of 330 μm before and 383 μm after the protocol, lacking evidence for fragmentation. These sizes correspond to the length of the fibers, while most widths were well below 10 μm. Fibers are not fully limited by PM_10_ inlet, crossing it as long as their shortest dimension was under 10 μm and they were correctly oriented. Similar behavior was reported by Abbasi et al. [7], who also used PM inlets and observed fibers of longer lengths. Moreover, 10 μm was the observed detection limit of this method, using the stereomicroscope (6x). Even though the main objective was the identification of microfibers, small particles (<10 μm) were also observed and likely comprised microplastics. These particles were quantified separately, since they were not fibers and their nature could not be fully assessed. The largest of these particles presented surface characteristics compatible with microplastics and should be submitted to chemical identification. Considering the diagram for the classification of fibers, and considering all fibers counted before and after the protocol, most were classified as natural (30 %) while a smaller proportion was identified as synthetic (6 %). The great majority of fibers could not be identified as either natural or synthetic (64 %), mostly due to the small dimensions presented (<250 μm) or low contrast with the filter in the case of light-colored fibers.

Blanks contained an average of 27 fibers during three transfers and two weeks of testing this protocol, being few compared to the hundreds of fibers detected in samples. Contamination found in blank samples are likely released from the cotton lab coat and paper towels used to maintain the laminal flow hood clean, with also some possible cross contamination from the filtration system. This type of contamination is almost impossible to avoid, taking into consideration the rigorous contamination prevention measures. An alternative is using lab coats and towels of strong recognizable colors which can help identify and exclude these fibers in environmental samples (*e.g.* green disposable lab coats used in medicine). The filtrations system cleaning between samples can be improved by dipping it in acid or other cleaning solution, additionally to the distilled water already used.

## Conclusion

The protocol previously developed by Abbasi et al. [7] for identification of microfibers and microplastics in deposited dust was adapted, improved and simplified for active sampling. These adaptations included removing weeklong periods of drying samples in 80 °C sandbathes, transferring samples by washing filters with ultrapure water or NaI, reducing the concentration of H_2_O_2_ used from 30 to 15 %, and proposing an improved identification diagram for fibers under the stereomicroscope. These improvements allowed to reduce the number of transfers and necessary time to finish the protocol (from 4 to 1.5 week), reducing the risk of contamination and improving sample throughput. The effects of the solutions used in common textile fibers demonstrate the innocuity of this protocol, which is also supported by the 94.4 % recovery rate of synthetic fibers and fragments. Thus, this protocol offers a simple and efficient method of treating air samples for the identification of microplastics and microfibers in the atmosphere.

## Declaration of Competing Interest

The authors declare no conflicts of interest.
