# Supplementary material for: An easy method for processing and identification of natural and synthetic microfibers and microplastics in indoor and outdoor air
Source: MethodsX. 2019 Dec 4;7:1–9. doi: 10.1016/j.mex.2019.11.032 (PMC6931130; doi:10.1016/j.mex.2019.11.032)
Supplement: Supplementary file 1 [file mmc1.docx]

**
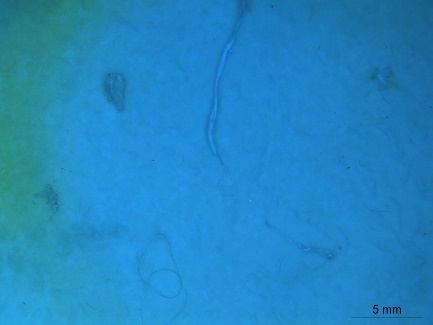

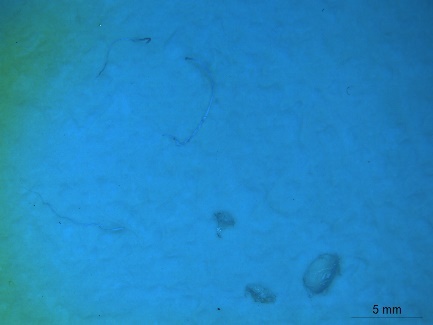

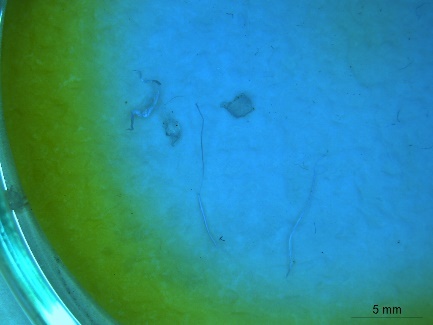
**

**Figure S-1.** Six fibers of polystyrene and six fragments of polyethylene were used as spikes and processed following the protocol. All particles were recovered apart for a polyethylene fragment that was found in inside a NaI beaker.
